# Gastrointestinal stromal tumor of the anal wall in a Nigerian

**DOI:** 10.11604/pamj.2015.22.161.8071

**Published:** 2015-10-20

**Authors:** Aderemi Oluyemi, Samuel Keshinro, Abimbola Jimoh, Philip Oshun

**Affiliations:** 1 ReMay Consultancy & Medical Services, Ikeja, Lagos, Nigeria; 2 MeCure Healthcare Limited, Oshodi, Lagos, Nigeria; 3 College of Medicine, University of Lagos, Nigeria

**Keywords:** Gastrointestinal stromal tumors (GIST), anal wall, immunohistochemistry, Nigeria

## Abstract

Documented reports of gastrointestinal stromal tumors (GIST) are relatively few in the sub-Saharan continent. The body of evidence points towards anal wall involvement being a rarity indeed. In this article we document a 61 year old Nigerian man who presented with bleeding per rectum and in whom the histological features (including immunohistochemistry) of the biopsied anal lesion was GIST.

## Introduction

Mazur and Clark were the first to coin the term “Gastrointestinal stromal tumors (GISTs)” in 1983 [1]. The pair of scientists characterized this group of tumors at the subcellular and molecular levels in detail and is so doing were able to demonstrate that GISTs are distinct entities from leiomyomas and leiomyosarcomas - the group that they had, hitherto, been lumped into. Overall, they are still relatively uncommon findings as they represent a mere 0.3 to 1% of gastrointestinal neoplasms [2]. The incidence of these relatively rare tumors is rising as more mesenchymal tumors are now being tested for the C-Kit protein (CD117) which is one of the characteristic immunological markers that these lesions express [3]. Immunohistochemistry is relatively unavailable in resource-limited countries like Nigeria. The absence of this vital tool for definitive diagnosis of GIST tumors has called into question many-a locally diagnosed “GIST” tumor as aptly demonstrated by Ezeome et al [4]. Two thirds of GISTs occur in the stomach while the small intestine GISTs account for two third of the others [2]. The rarity of anal wall site is well documented as only a handful has been reported [5]. This article seeks to document what, to the best of our knowledge, would be the first such immunohistochemically proven case of GIST of the anal wall from our region.

## Patient and observation

**Figure 1:**
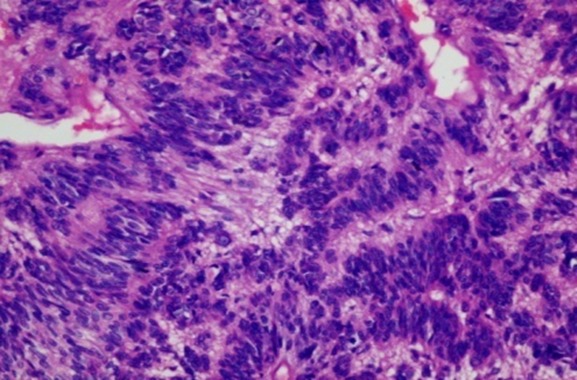
Low power photomicrograph of the anal lesion. It shows the necrotic tumor comprising of proliferating stromal cells with peripheral palisading (Hematoxylin and Eosin stain; original magnification × 40)

**Figure 2:**
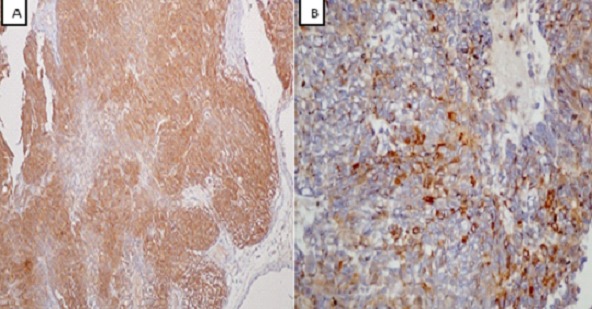
(A): Immunohistochemical stain patterns showing strong positivity for c-kit; (B): and moderate positivity for SMA (original magnification × 40)

**Figure 3:**
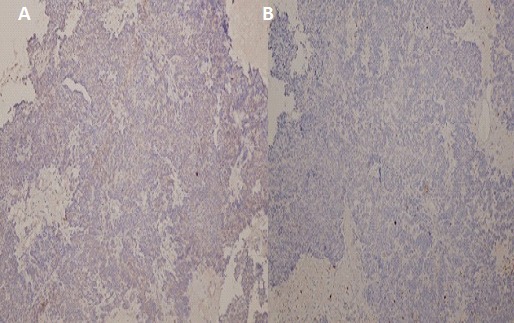
(A): Immunohistochemical stain patterns showing negativity for Pan CK; (B): and protein S 100 (original magnification × 40)

**Figure 4:**
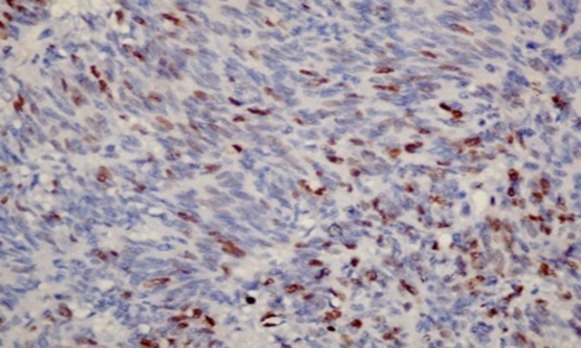
Immunohistochemistry for Ki-67 showing some nuclear positivity (original magnification × 40)

A 61 year old Nigerian gentleman was referred for colonoscopy in Lagos, Nigeria on account of passage of blood per rectum. The patient had noted the initial episode about 3 months prior to presentation. The blood was noted to precede and also line the periphery of the fecal bulk that was expelled. There was no history of alteration in bowel habit nor did the patient report any other systemic symptoms. The patient was in stable clinical condition on examination and the digital rectal examination had revealed a small, soft mass in the anterior aspect of the anal wall with the withdrawn finger being stained with blood. A packed cell volume test was 41%. Upon retroflexion in the rectum, the colonoscopy findings were that of a 5 cm in diameter polypoidal mass in the anterior wall of the anal canal. The mass had multiple umbilications on its irregular surface and was noted to be gently oozing blood at the time of examination. Cold forceps were used to biopsy the lesion and samples were sent for histology. The clinical suspicion of a malignant tumour was raised. An abdominal computed tomography (CT) scan revealed a small mass in the anal wall with focal mural thickening and minimal fat stranding which is indicative of limited local infiltration. There was no radiographic suggestion of regional lymph node involvement. The microscopic examination showed complete replacement of the tissue by a focally necrotic tumor that comprised proliferating stromal cells with peripheral palisading (Figure 1). Nuclear atypia was absent and mitotic count was 5 per 10 high Power fields. The immunohistochemical stain patterns showed strong positivity for C-kit (CD 117), moderate positivity for smooth muscle antigen (SMA) and weak positivity for CD 34. Other immunohistochemical staining patterns reveal Ki-67 (6-8%) while protein S-100 and Pan CK were negative (Figure 2, Figure 3, Figure 4). The combination of the two modalities confirmed the absence of carcinoma and the strong positivity for CD 117 indicated the lesion was a GIST. Upon review of the results, the findings were communicated to the referring physician and the patient was then sent to a Gastroenterologist closer to his base. The patient is said to have had surgery for the mass and has already been commenced on imatinib.

## Discussion

The above case report details a rare case of GIST of the anal wall in an elderly Nigerian patient. The case, with its full immunohistochemical characterization, to the best of the authors’ knowledge, is the first of such from this region of Africa. The occurrence of anal GIST is very rare indeed as it represents only 3% of ano-rectal mesenchymal tumors [6]. Put in perspective, GISTs only occur in this region in 5% of cases [7]. In fact, a 2007 review found only ten cases of c-kit positive anal GIST in the scientific literature [8]. GIST is not a new entity to this part of the world as several local case reports and series have examined various aspects of the tumor [4]. Immunohistochemistry has been noted to be crucial for definitive diagnosis but this important component has not always featured in many-a-locally made diagnosis [4, 9, 10]. The obvious reason for this is that the modality is not readily available in our environment [4, 11]. The case here presented highlights this area of need in gastroenterology in this locality as the immunohistochemistry was done in a foreign center. Prognostic indices have been well characterized in GIST and include the size of the tumor, the mitotic index of the lesion, as well as some as yet undefined malignant factors as acknowledged in the 2002 consensus guidelines [3]. These undetermined factors have also instructed the wordings of the guidelines to highlight that GIST is categorized in risk groups, further emphasizing that no lesion is definitely labeled as “benign” [3]. The therapeutic approach in this case is interesting. The surgical team decided to progress to abdominoperineal resection as the pre-surgical CT scan was positive for local infiltration though negative for regional lymph node involvement. His advanced age notwithstanding, the decision for surgery was made as the risks of GIST-related morbidity (and possible mortality) with only excision was unacceptable. This was in keeping with sound scientific opinion from literature [8]. The role of tyrose kinase inhibitors, imatinib in this case, is still controversial. But the surgical team planned to administer this to the patient for at least one year and continue lifelong monitoring in view of possibility of recurrences or metastases [6]. The first year post follow up screens done at the patient's base (CT scan and colonoscopy with biopsy) were all normal and patient was said to be in good health.

## Conclusion

The report documents a rare case of anal GIST as a cause of hematochezia in a patient from sub-Saharan Africa. The need for advanced histopathologic assessment (including detailed immunohistochemitry) of all such lesions to provide further clarity after basic histology stains is further emphasized- even as we note that the resource constrained environment that we live in makes this easier said than done.
